# Metabolic dysfunction-associated steatotic liver disease: Pathogenesis, complications and pharmacological treatment

**DOI:** 10.17179/excli2026-9310

**Published:** 2026-09-22

**Authors:** Kyung-Soo Kim, Soo Lim, Cheol-Young Park

**Affiliations:** 1 Department of Internal Medicine, CHA Bundang Medical Center, CHA University School of Medicine, Seongnam, South Korea; 2 Department of Internal Medicine, Seoul National University College of Medicine, Seoul National University Bundang Hospital, Seongnam, South Korea; 3 Division of Endocrinology and Metabolism, Department of Internal Medicine, Kangbuk Samsung Hospital, Sungkyunkwan University School of Medicine, Seoul, South Korea

**Keywords:** cardiovascular diseases, fatty liver, hepatocellular carcinoma, liver cirrhosis, metabolic dysfunction-associated steatohepatitis, nonalcoholic fatty liver disease, semaglutide, steatosis of liver, type 2 diabetes mellitus

## Abstract

Metabolic dysfunction-associated steatotic liver disease (MASLD) is increasing in prevalence worldwide. Pathogenesis of MASLD has been considerably heterogenous. Increased hepatic de novo lipogenesis, adipose tissue dysfunction, genetic predisposition, epigenetic mechanism, and other related factors have been associated with the development and progression of MASLD. Patients with MASLD are at high risk of not only adverse liver-related outcomes, including hepatocellular carcinoma, but also cardiometabolic disease including type 2 diabetes mellitus. Weight loss is the cornerstone of therapy among the various treatments for MASLD to decrease hepatic fat accumulation and delay the progression of inflammation and fibrosis. Resmetirom and semaglutide are approved for the treatment of metabolic dysfunction-associated steatohepatitis with moderate to advanced fibrosis, and many drugs are under investigation. Careful attention should be paid to the screening of MASLD, and concerted efforts should be made to manage it appropriately.

See also the graphical abstract(Fig. 1).

## Introduction

Metabolic dysfunction-associated steatotic liver disease (MASLD) is an escalating epidemic that is tied to the surge in obesity and diabetes and has steadily increased worldwide (Riazi et al., 2022[109]; Younossi et al., 2023[161]). The terms MASLD and metabolic dysfunction-associated steatohepatitis (MASH) were introduced in 2023 to replace non-alcoholic fatty liver disease (NAFLD) and non-alcoholic steatohepatitis (NASH), respectively (Rinella et al., 2023[111]), and can be applied to populations previously classified under NAFLD and NASH (Hagstrom et al., 2024[47]; Ratziu et al., 2024[108]; Song et al., 2024[131]). The MASLD spectrum encompasses simple steatotic liver disease (SLD) to MASH, progressive fibrosis, and ultimately cirrhosis, leading eventually to hepatocellular carcinoma (HCC) (Sheka et al., 2020[124]). The prevalence of MASLD in adult populations varies according to different diagnostic criteria, but is now estimated at 38 % (Stefan et al., 2025[135]). Notably, MASLD is highly prevalent among individuals with type 2 diabetes mellitus (T2DM), affecting approximately 60-80 % of this population (En Li Cho et al., 2023[36]; Lee et al., 2019[71]).

Approximately 20 % of patients with MASLD have their condition progress to MASH, and 20 % of those with MASH have their condition progress to cirrhosis, though some patient conditions can be reversible (Sheka et al., 2020[124]). In addition, patients with MASLD are at high risk of not only adverse liver-related outcomes, including HCC, but also extrahepatic outcomes, such as cancer and cardiometabolic disease (Cho et al., 2019[26]; Lim et al., 2019[76]). Cardiovascular disease (CVD) is already one of the leading causes of death in patients with MASL (Adams et al., 2005[2]; Ong et al., 2008[96]). There is no doubt that appropriate management of MASLD patients is crucial for global health care. This article aims to provide an in-depth review of pathogenesis, complications and implications for diagnosis and treatment of MASLD.

## Pathogenesis

Excess accumulation of lipids in the liver is a fundamental process in the development and progression of MASLD (Lauridsen et al., 2018[69]; Shulman, 2014[125]; Tchkonia et al., 2013[144]). Excessive intake of fatty acids contributes directly to hepatic triglyceride accumulation, whereas excessive intake of glucose and fructose promotes triglyceride synthesis through de novo lipogenesis in the liver (Roden and Shulman, 2019[113]; Yki-Järvinen et al., 2021[160]). Excessive fatty acids in the body results in subclinical inflammation in adipose tissue and the liver, and insulin resistance in adipose tissue, liver, and skeletal muscle (Korenblat et al., 2008[67]). Increased signaling via fatty acids, diacylglycerols, ceramides, and various factors are involved in the process, the resulting insulin resistance-associated hyperinsulinemia and hyperglycemia amplify hepatic de novo lipogenesis (Bo et al., 2024[17]; Gastaldelli and Cusi, 2019[42]). In addition, hepatic mitochondrial dysfunction, increased oxidative stress, and uncoupling of oxidative phosphorylation are also promoted by increased saturated fatty acids and hyperglycemia (Johnson et al., 2024[57]). This process causes cell stress and macrophage activation, resulting in a fibrogenic response in hepatic stellate cells that can promote the progression to cirrhosis (Friedman et al., 2018[39]; Loomba et al., 2021[79]). Dysregulated release of adipokines and cytokines from adipose tissue dysfunction also might be the predominant pathogenic mechanism in MASLD (Kahn et al., 2019[58]). There is growing evidence that genetic predisposition is associated with an increased risk of hepatic steatosis, inflammation, fibrosis, and HCC (Ajmera and Loomba, 2023[5]; Luukkonen et al., 2023[81]; Romeo et al., 2020[115]).

### Increased hepatic de novo lipogenesis

Increased hepatic de novo lipogenesis is a main contributor to hepatic steatosis. A study showed that 26 % of the hepatic triglycerides are derived from de novo lipogenesis in patients with MASLD (Donnelly et al., 2005[34]). In the study of 67 subjects, the contribution of hepatic de novo lipogenesis to intrahepatic triglyceride palmitate was 11 %, 19 %, and 38 % in the lean, obese, and obese-NAFLD groups, respectively (Smith et al., 2020[127]). It means that hepatic de novo lipogenesis is an important contributor to intrahepatic triglyceride production and intrahepatic triglyceride content in individuals with MASLD. In addition, the contribution of hepatic de novo lipogenesis to intrahepatic triglyceride was negatively correlated with both hepatic and whole-body insulin sensitivity and positively correlated with integrated 24-hour plasma glucose and insulin concentrations. In an analysis of data from 37,358 UK Biobank participants, all liver triglyceride content-raising variants were associated with increased risk of non-alcohol-related cirrhosis, HCC, and intrahepatic bile duct and gallbladder cancers (Ahmed et al., 2024[3]). Interestingly, genetic variants associated with enhanced de novo lipogenesis and higher liver triglyceride content were linked to a higher risk of myocardial infarction and coronary artery disease, whereas genetic variants associated with impaired hepatic triglyceride export and higher liver triglyceride content were linked to a lower risk of coronary artery disease and myocardial infarction.

### Adipose tissue dysfunction

Excessive delivery of fatty acids to the liver caused by dysregulated lipolysis in adipose tissue is also an important contributor to hepatic steatosis. Because insulin is a primary suppressor of adipocyte lipolysis, adipose tissue insulin resistance contributes to dysregulated lipolysis. Adipose tissue dysfunction might be the predominant pathogenic mechanism in patients with lean MASLD. Dietary oversupply of fat and carbohydrates causes a stress response in adipose tissue that impairs insulin signaling and reduces beneficial adipokine production such as adiponectin (Ghaben and Scherer, 2019[44]; Kahn et al., 2019[58]; Karpe and Pinnick, 2015[60]; Stefan, 2020[133]; Stefan et al., 2017[134]; Tchkonia et al., 2013[144]). Patients with MASLD have lower serum adiponectin levels, which are associated with greater systemic insulin resistance, endothelial dysfunction, accelerated atherosclerosis, and higher risk of adverse CVD outcomes (Polyzos et al., 2016[102]). Additionally, treatment with adiponectin was shown to enhance insulin-mediated glucose uptake while inhibiting hepatic gluconeogenesis in mouse models (Miller et al., 2011[88]) and improved hepatic fat accumulation in an animal model of diabetes (Long et al., 2014[78]).

### Genetic predisposition

Genetic predisposition derived from genome-wide association and exome sequencing studies might contribute to the underlying metabolic abnormalities leading to MAFLD (Cotter and Rinella, 2020[28]). The identified genetic variants and the associated altered pathways are often involved in the regulation of the mobilisation of triglycerides from lipid droplets (*PNPLA3*), assembly and secretion of VLDLs (*TM6SF2*), hepatic phosphatidylinositol acylchain remodeling (*MBOAT7*), or de novo lipogenesis (*GCKR*) (Ajmera and Loomba, 2023[5]; Romeo et al., 2020[115]). Multiple studies have reported that patients with MASLD based on a strong hepatic genetic component have an increased risk of hepatic steatosis, inflammation, fibrosis, and HCC. MASLD-risk allele were found to have hepatic mitochondrial dysfunction leading to reduced de novo lipogenesis and channeling of carbons to ketogenesis in a study of people homozygous for the *PNPLA3* Ile148Met (*PNPLA3* rs738409 G) (Luukkonen et al., 2023[81]). In a large exome-wide association study of plasma lipids, the *PNPLA3* rs738409 G MASLD-risk allele and the *TM6SF2* rs58542926 T MASLD-risk allele were strongly associated with steatosis and progression to MASH, cirrhosis, and HCC (Lauridsen et al., 2018[69]). Especially, PNPLA3 was identified as a triglyceride lipase mobilizing polyunsaturated fatty acid to facilitate hepatic secretion of large-sized very low-density lipoprotein. The *PNPLA3* rs738409 G MASLD-risk allele was less active than the wildtype enzyme, indicating that hepatic steatosis might result from decreased hepatic triglyceride secretion (Johnson et al., 2024[57]).

### Epigenetic mechanism

Epigenetics affecting gene expression without altering DNA sequences is also one of MASLD pathophysiology. Epigenetic regulation is essential for maintaining hepatic metabolic function and cellular identity in response to metabolic cues but becomes critically dysregulated during MASLD (Pearce et al., 1992[99]). Environmental factors (diet, obesity, and aging) can induce epigenetic changes that drive MASLD progression. DNA methylation processes, histone modifications, and microRNAs (miRNAs) expression have been closely associated with MAFLD progression (Pirola and Sookoian, 2022[100]; Wu et al., 2023[156]).

DNA methylation is the most studied epigenetic mechanism in MASLD (Rodriguez-Sanabria et al., 2021[114]). A study found that 41 genes responsible for lipid homeostasis were significantly and differentially methylated, including members of the APO family (lipid transport), genes involved in cholesterol transport like intracellular cholesterol transporter 1 (*NPC1L1*), acyl-CoA, sterol regulatory element binding transcription factor 1 (*SREBF1*), StAR-related lipid transfer domain containing 5 (*STARD5*), and solute carrier family 2 member 4 (*SLC2A4*) (Mwinyi et al., 2017[93]). In addition, an increased hepatic methylation of peroxisome proliferator-activated receptor gamma coactivator-1 alpha (*PPARGC1A*) has been correlated with high plasma fasting insulin levels and HOMA-IR in patients with MASLD (Santos et al., 2020[117]; Sookoian et al., 2010[132]).

Histones are the basic structural proteins of chromosomes, rich in alkaline amino acid (arginine and lysine), which can facilitate the binding of acid DNA. Many histone posttranslation modifications are confirmed, such as phosphorylation (e.g., H3K9me3 and H3K36me3) (Chen et al., 2024[24]; Oki et al., 2007[95]), methylation (e.g., H3K27me3, H3K79me2/3, H3K4me1, H3K9me3, H3K36me2/3, H3R8me2, and H4K20me3) (Chen et al., 2024[24]; Dimitrova et al., 2015[32]), ubiquitylation (e.g., H2Bub1, H2AK119ub, H2AK121ub, H2BK120ub, and H2BK123ub) (Chen et al., 2024[24]; Wang et al., 2004[151]), acetylation (e.g., H3K9ac, H3K14ac, H3K23ac, and H3K27ac) (Chen et al., 2024[24]; Xia et al., 2022[157]), lactylation (Lv et al., 2023[82]), and crotonylation (Tan et al., 2011[138]), which make up the histone code.

MicroRNAs (miRNAs) are single-stranded non-coding RNAs of 18-25 nucleotides that can regulate gene expression at posttranscriptional level by inhibiting translation or inducing degradation of target mRNAs through complementary base-pairing (Bartel, 2004[14]). Evidence has demonstrated that miRNAs are implicated as important mediators in MASLD (Baffy, 2015[11]; Szabo and Csak, 2016[137]). Several miRNAs including miR-27b, miR-33, miR-34a, miR-122, and miR-223 are important regulators in fatty acid metabolism and cholesterol biosynthesis in the liver (Landrier et al., 2019[68]).

## Complications

### Liver-related outcomes

The histological characteristics of MASLD range from mild steatosis to MASH, which, if not addressed, can cause fibrosis, cirrhosis, and HCC (Chalasani et al., 2018[22]). In general, people with MASLD are at an increased risk of developing cirrhosis and HCC, and in approximately 15 % to 20 % of those with MASH, progress to cirrhosis, HCC, and liver death is observed (Powell et al., 2021[103]; Sheka et al., 2020[124]; Younossi et al., 2023[163]). In a prospective study of 1,773 adults with MASLD, fibrosis stages F3 (bridging fibrosis) and F4 (cirrhosis) were associated with increased risks of liver-related complications and death (Sanyal et al., 2021[123]). In a meta-analysis of 13 studies comprising 4,428 patients with MASLD, patients with stage 4 fibrosis (cirrhosis) had higher liver-related mortality (relative risk [RR] 11.13, 95 % CI 4.15-29.84) than those without fibrosis (Taylor et al., 2020[143]). This risk is potentiated in patients with both MASLD and T2DM. A meta-analysis of 2,016 patients of six cohorts, among people with MASLD, the presence of T2DM is associated with a significantly higher risk of hepatic decompensation (hazard ratio [HR] 3.29, 95 % CI 2.21-4.90) and HCC (HR 7.72, 95 % CI 2.61-22.87), compared with patients without diabetes (Huang et al., 2023[53]).

### Pathophysiologic link between MASLD and T2DM

MASLD and T2DM are mechanistically linked through a bidirectional and self-reinforcing network of insulin resistance, lipotoxicity, inflammation, and β-cell stress (Figure 2(Fig. 2)). Upstream, adipose tissue insulin resistance driven by adipocyte hypertrophy and hypoxia enhances lipolysis and increases free fatty acid flux to the liver, accompanied by adipokine imbalance characterized by increased TNF-α and IL-6 and reduced adiponectin, thereby promoting systemic and hepatic insulin resistance (Erceg et al., 2025[37]; Mantovani et al., 2018[83]). In parallel, skeletal muscle insulin resistance impairs glucose uptake and increases hepatic substrate delivery, contributing to persistent hyperglycaemia and loss of metabolic flexibility (Mohiuddin et al., 2025[90]). Within the liver, excess lipid availability stimulates SREBP-1c-mediated de novo lipogenesis and accumulation of lipotoxic intermediates such as diacylglycerols and ceramides, leading to PKCε activation, impaired insulin signaling, increased hepatic gluconeogenesis, and enhanced VLDL secretion-core mechanisms underlying fasting and postprandial dysglycaemia (Chung et al., 2026[27]; Mohiuddin et al., 2025[90]). Hepatic oxidative stress and activation of inflammatory pathways, including NLRP3 inflammasome signaling, further amplify hepatic and systemic insulin resistance (Erceg et al., 2025[37]). As illustrated in Figure 2(Fig. 2), disruption of the gut-liver-pancreas axis provides an additional mechanistic link, whereby gut dysbiosis, reduced short-chain fatty acid production, increased intestinal permeability, and translocation of microbial products impair incretin signaling and exacerbate insulin resistance and β-cell stress (Guo et al., 2025[45]; Jing and Jiang, 2025[56]; Liang et al., 2025[74]; Tilg et al., 2026[147]; Zhou et al., 2026[166]). Downstream, chronic hyperglycaemia and compensatory hyperinsulinaemia induce glucolipotoxic stress on pancreatic β-cells, ultimately leading to β-cell dysfunction and failure, while established T2DM reciprocally accelerates MASLD progression. Consistent with this integrated pathophysiologic framework, epidemiologic studies demonstrate that MASLD is independently associated with incident T2DM, with risk increasing in parallel with hepatic disease severity and fibrosis burden (Chung et al., 2026[27]; Han et al., 2025[48]; Mantovani et al., 2018[83]).

Mechanistic and clinical evidence support that T2DM is bidirectionally related to MASLD (Lim et al., 2019[76]; Mohiuddin et al., 2025[90]; Targher et al., 2021[141]). MASLD is a very common condition among patients with T2DM, and individuals with MASLD have an increased risk of developing T2DM (Bril and Cusi, 2016[18]). In a meta-analysis of 501,022 adult individuals (30.8 % with MASLD), MASLD was associated with 2.2 times higher risk of incident T2DM over a median period of 5 years (Mantovani et al., 2020[86]). Patients with T2DM are more likely to develop advanced fibrosis, cirrhosis, and HCC than those without T2DM (Anstee et al., 2013[9]). Furthermore, MASLD and T2DM have a synergistic association that contributes to cardiovascular risk. In a nationwide, population based, longitudinal cohort study with 7,796,763 Koreans, MASLD in patients with T2DM was associated with a higher risk of CVD and all cause death, even in patients with mild MASLD (Kim et al., 2024[63]). Although the exact mechanisms linking MASLD with CVD in T2DM patients are not fully understood, they likely involve distinct pathophysiological pathways. Specifically, these pathways encompass low-grade chronic inflammation, proatherogenic lipid profiles (such as increased very low density lipoprotein and small, dense low density lipoprotein cholesterol), and prothrombotic states driven by altered coagulation factors and platelet reactivity (Biondi-Zoccai et al., 2003[16]; Brouwers et al., 2020[19]; Santos et al., 2019[118]). The American Diabetes Association now recommends to “Screen adults with T2DM or with prediabetes, particularly those with obesity or other cardiometabolic risk factors or established CVD, for their risk of having or developing cirrhosis related to MASH using a calculated fibrosis-4 index (FIB-4), even if they have normal liver enzymes” (American Diabetes Association Professional Practice Committee for Diabetes, 2026[7]).

### Pathophysiologic link between MASLD and CVD

MASLD is increasingly recognized as an active driver of CVD, rather than a passive marker of shared cardiometabolic risk. As illustrated in Figure 3(Fig. 3), adipose tissue insulin resistance represents an upstream trigger, promoting enhanced lipolysis and increased free fatty acid flux to the liver, together with adipokine imbalance characterized by reduced adiponectin and elevated pro-inflammatory cytokines such as TNF-α and IL-6 (Targher et al., 2024[140]; Tilg and Moschen, 2010[146]). These processes fuel hepatic steatosis through maladaptive de novo lipogenesis and accumulation of lipotoxic intermediates, including diacylglycerols and ceramides, which induce hepatic insulin resistance, mitochondrial dysfunction, and oxidative stress (Friedman et al., 2018[39]). Concomitantly, activation of innate immune pathways-particularly TLR4-dependent signaling and NLRP3 inflammasome activation-amplifies hepatic and systemic inflammation, leading to increased circulating levels of IL-1β, IL-6, and C-reactive protein, thereby linking hepatic injury to vascular inflammation (Lim et al., 2019[76]; Mantovani et al., 2021[85]). As depicted in Figure 3(Fig. 3), endothelial dysfunction emerges as a critical downstream consequence, driven by reduced nitric oxide bioavailability, heightened oxidative stress, and increased vascular tone, collectively fostering a pro-atherogenic environment that accelerates monocyte recruitment, foam-cell formation, and plaque progression (Abohashem et al., 2026[1]; Ratti et al., 2025[107]; Targher et al., 2016[139]; Wang et al., 2024[154]). Alterations in the gut-liver axis, including dysbiosis and endotoxin translocation, further reinforce these inflammatory circuits (Friedman et al., 2018[39]; Pandey et al., 2025[97]; Wang et al., 2025[152]). Consistent with this integrated pathophysiologic framework, epidemiologic and imaging studies demonstrate that MASLD is independently associated with subclinical atherosclerosis and adverse cardiovascular outcomes, with CVD risk increasing in parallel with disease severity and fibrosis stage (Han et al., 2025[48]; Mantovani et al., 2022[87]; Targher et al., 2021[141]).

Notably, CVD is one of the leading causes of death in patients with MASLD (Adams et al., 2005[2]; Ong et al., 2008[96]). In a population-based cohort study with a 28-year follow-up, MASLD predicted the risk of adverse CVD events, independent of common cardiovascular risk factors (Pisto et al., 2014[101]). A systematic review and meta-analysis of 26 observational studies with 85,395 participants showed that patients with MASLD had a higher risk of subclinical atherosclerosis than those in the non-MASLD group (odds ratio [OR] 1.60, 95 % confidence interval [CI] 1.45 to 1.78) (Zhou et al., 2018[167]). In a meta-analysis of 36 longitudinal studies with aggregate data on 5,802,226 middle aged participants, MASLD has been associated with an increased long-term risk of fatal or non-fatal CVD events (Mantovani et al., 2021[85]). Furthermore, the fibrosis stage was also associated with long-term adverse outcomes in patients with MASLD (Angulo et al., 2015[8]).

### Association between MASLD and mortality

Accumulating population-based evidence demonstrates that MASLD is independently associated with increased long-term mortality, particularly in the presence of metabolic risk factors and advanced fibrosis. Analyses from NHANES III with extended follow-up show that MASLD is associated with a significantly higher risk of all-cause mortality after adjustment for sociodemographic and lifestyle factors, whereas NAFLD defined by exclusion criteria alone does not retain an independent association once metabolic confounders are considered (Kim et al., 2021[61]; Song et al., 2024[130]). Excess mortality in MASLD is largely driven by cardiometabolic causes, with cardiovascular disease representing the leading contributor (Kim et al., 2021[61]). Consistent findings across U.S. and East Asian cohorts, including nationwide studies from Korea and Taiwan, demonstrate a 15-30 % increase in all-cause mortality among individuals with MAFLD/MASLD, with particularly elevated risk observed in those meeting MASLD but not NAFLD criteria (Cheng et al., 2024[25]; Kim et al., 2023[62]; Moon et al., 2022[91]; Song et al., 2024[130]). Across studies, advanced fibrosis emerges as the strongest determinant of mortality, conferring an approximately 1.7-2-fold increase in death risk irrespective of disease nomenclature (Kim et al., 2021[61]; Moon et al., 2022[91]; Song et al., 2024[130]). Collectively, these data support MASLD as a clinically meaningful construct that captures mortality risk in various ethnic groups.

## Diagnosis

MASLD is diagnosed by the presence of hepatic steatosis in conjunction with cardiometabolic risk factors, reflecting a shift from exclusion-based definitions toward a positive, pathophysiology-oriented framework. According to international consensus, MASLD requires evidence of liver steatosis-identified by imaging modalities such as ultrasonography, controlled attenuation parameter, or MRI-proton density fat fraction, or by histology-together with at least one cardiometabolic criterion, including overweight or obesity, T2DM, or defined metabolic abnormalities (Rinella et al., 2023[111]; Targher et al., 2025[142]). Importantly, MASLD may coexist with other liver diseases, acknowledging the frequent overlap between metabolic dysfunction and alternative hepatic insults (Targher et al., 2025[142]).

Although not included in the formal diagnostic criteria, surrogate indices such as the fatty liver index (FLI) have been widely used to identify hepatic steatosis in epidemiologic and population-based studies (Kim et al., 2024[63]). FLI is calculated as

FLI = (e^0.953×ln(triglyceride)+0.139×BMI+0.718×ln(^^γ-glutamyl transferase)+0.053×waist circumference−15.745^) ÷ (1+e^0.953×ln(triglyceride)+0.139×BMI+0.718×ln(γ-glutamyl transferase)+0.053×waist circumference−15.745^) × 100 (1)

and has been validated as a simple and accurate marker of hepatic steatosis across diverse populations (Bedogni et al., 2006[15]; Cuthbertson et al., 2014[30]; Huang et al., 2015[54]). In general, an FLI ≥ 60 reliably identifies hepatic steatosis, whereas lower thresholds (e.g., ≥ 30) are commonly applied in Asian populations (Ballestri et al., 2021[12]; Bedogni et al., 2006[15]; Huang et al., 2015[54]).

Because prognosis and management of MASLD are largely determined by fibrosis severity, noninvasive fibrosis assessment constitutes a central component of the diagnostic pathway (Castera et al., 2025[21]). Widely validated blood-based tools include the aspartate aminotransferase-platelet ratio index (APRI), FIB-4, and the Enhanced Liver Fibrosis (ELF) score (Rosenberg et al., 2004[116]; Sterling et al., 2006[136]; Wai et al., 2003[150]). The FIB-4 score is calculated as FIB-4=(age×AST level)÷(platelet count×√ALT level) (2), and represents the most cost-effective first-line screening strategy for identifying individuals with clinically significant fibrosis or at-risk MASH in primary care and diabetes settings (American Diabetes Association Professional Practice Committee for Diabetes, 2026[7]). A FIB-4 value ≥ 1.3 indicates increased probability of fibrosis stage ≥ F2 and heightened risk of adverse liver outcomes, warranting further evaluation with elastography-based techniques (Castera et al., 2025[21]). Emerging evidence further supports incorporation of extrahepatic features such as sarcopenia and altered body composition into diagnostic assessment, as these factors identify clinically vulnerable MASLD phenotypes not fully captured by liver-centered metrics alone (Liu et al., 2026[77]).

## Treatment

In patients with MASLD, weight loss is the treatment of choice to decrease hepatic fat accumulation and delay the progression of inflammation and fibrosis (Lazo et al., 2011[70]). Although there is significant individual variability in histological outcomes with weight loss, a minimum weight loss goal of 5 %, preferably ≥ 10 %, is needed to improve liver histology, with fibrosis requiring the larger weight reduction to promote change (Cusi et al., 2022[29]; European Association for the Study of the Liver et al., 2024[38]; Kanwal et al., 2021[59]; Promrat et al., 2010[104]; Rinella et al., 2023[112]; Vilar-Gomez et al., 2015[148]). Of the ways to achieve weight reduction, lifestyle modifications including a programmed diet and exercise are effective and sound treatment options for all patients with MASLD and MASH (Kim et al., 2019[65]; Rinella, 2015[110]) (Figure 4(Fig. 4)). Individualized, structured weight loss and exercise programs may offer greater benefit than standard counseling in people with MASLD (Younossi et al., 2023[163]). A systematic review of 23 studies of adult populations with MASLD revealed that weight loss by lifestyle modification was the most strongly correlated with reductions in liver fat and/or liver aminotransferase concentrations (Thoma et al., 2012[145]).

Lifestyle modification in MASLD is focused on behavior change to support sustained beneficial lifestyle practices such as improving overall dietary patterns (low fat and calorie diet or Mediterranean type of diets), moderate caloric restriction and regular physical activity (Wadden et al., 2020[149]). Lifestyle modification is best achieved through structured, multi-disciplinary programs with ongoing support to facilitate sustained behavior change (Alamuddin and Wadden, 2016[6]). From a dietary standpoint, key elements include a balanced diet with limited intake of simple carbohydrates and saturated fat, and higher intake of lean proteins, whole grains, fruits and vegetables, and omega-3 fatty acids (Lichtenstein et al., 2021[75]). The Mediterranean diet demonstrates particularly potent anti-inflammatory effects.

Physical activity has a significant positive impact on MASLD. In randomized trials, aerobic physical activity improves several pathogenetic factors in MASLD, including inflammation and endothelial dysfunction (Wewege et al., 2018[155]). Furthermore, the addition of resistance training to aerobic activity is linked to further improvements in MASLD and is therefore advised as part of the exercise regimen (Xue et al., 2024[158]). In general, moderate to vigorous physical activity on most days of the week is recommended, with a weekly goal of 150 minutes or more of physical activity per week (Barone Gibbs et al., 2021[13]). While weight loss with lifestyle modification is desirable, it is important for patients and healthcare professionals to recognize that improved dietary patterns and regular physical activity improve metabolic impairment independent of weight loss (Rao et al., 2019[106]). In addition to lifestyle modification, moderation of alcohol use should be advised to lessen the risk of hepatic complications.

### Pharmacological treatment

Although weight reduction is the cornerstone of therapy among the various treatments for MASLD, pharmacotherapy should be administered since sufficient weight reduction can be difficult to achieve and maintain (Kim and Lee, 2020[64]). The US Food and Drug Administration (FDA) published a whitepaper in 2011 detailing the roadmap of drug approval for the treatment of MASH, stating that for conditional approval, at least one of the following histologic endpoints should be reached: resolution of MASH (defined as an inflammation score of 0 or 1 and a ballooning score of 0) without worsening of fibrosis or improvement in fibrosis by one stage or more, without worsening of MASH (Sanyal et al., 2011[120]). The European Medicine Agency (EMA) requires both the resolution of MASH and improvement in fibrosis as the primary endpoint (Stefan et al., 2025[135]). As of early 2026, resmetirom is approved by both the US FDA and EMA for the treatment of MASH with moderate to advanced fibrosis and semaglutide is approved by US FDA (Table 1(Tab. 1)).

### Resmetirom

Thyroid hormone receptor (THR)-β is predominantly expressed in hepatocytes and plays a vital role in various metabolic processes (Sinha et al., 2019[126]). Activation of THR-β is associated with significant metabolic benefits in the liver because it can lower cholesterol and triglyceride levels, stimulate bile acid synthesis, and promote fat oxidation (Kokkorakis et al., 2024[66]). Resmetirom is a THR-β agonist approved by the US FDA for the treatment of adults with MASLD with moderate (F2) or advanced (F3) liver fibrosis on liver histology or a validated imaging- or blood-based test. In an ongoing phase 3 randomized controlled trial (RCT) (MAESTRO-NASH trial), resmetirom for 52 weeks in 966 adults at the highest dose of 100 mg (or placebo) met the primary end point of MASH resolution without worsening of fibrosis in 29.9 % of individuals compared with 9.7 % on placebo (P < 0.001) (Harrison et al., 2024[49]). Fibrosis improvement by at least one stage without worsening of the NAFLD Activity Score was seen in 25.9 % in the resmetirom 100 mg group compared with 14.2 % in the placebo group. The gastrointestinal side effects including nausea, vomiting, and diarrhea are dose dependent and improve with continued treatment. Because resmetirom could decrease free thyroxine levels by ∼20 % and increase sex hormone-binding protein levels two- to threefold, clinicians should monitor all individuals with MASLD for symptoms of endocrine deficiency, including hypothyroidism and hypogonadism (Chen et al., 2025[23]).

### Semaglutide

Glucagon-like peptide-1 (GLP-1) is released by enteroendocrine cells in the gastrointestinal tract to play a crucial role in regulating postmeal blood sugar levels by enhancing insulin secretion and suppressing glucagon release (Drucker, 2018[35]; Son et al., 2026[128]). GLP-1 also delays gastric emptying and reduces food intake, striking a balance between nutrient absorption and preventing excessive weight gain. GLP-1 receptor agonists are appealing candidates for the treatment of MASLD and MASH because they can reduce weight and enhance insulin action. Many preclinical studies found that these agonists could alleviate MASH by mitigating hepatic steatosis, inflammation, and injury, whereas these effects were suggested to be secondary to the weight loss induced by GLP-1 receptor agonists (Zhang et al., 2025[165]). Among various GLP-1 receptor agonists, semaglutide has shown encouraging outcomes in resolving MASH. In an ongoing phase 3 clinical trial examining the effect of semaglutide (2.4 mg subcutaneously once weekly for 72 weeks) in subjects with MASH without cirrhosis (ESSENCE trial), resolution of steatohepatitis without worsening of fibrosis occurred in 62.9 % of the 534 patients in the semaglutide group and in 34.3 % of the 266 patients in the placebo group (estimated difference, 28.7 percentage points; 95 % CI, 21.1 to 36.2; P < 0.001). A reduction in liver fibrosis without worsening of steatohepatitis was reported in 36.8 % of the patients in the semaglutide group and in 22.4 % of those in the placebo group (estimated difference, 14.4 percentage points; 95 % CI, 7.5 to 21.3; P < 0.001) (Sanyal et al., 2025[122]). Based on these phase 3 trial results, the US FDA approved semaglutide for the treatment of MASH with moderate to advanced liver fibrosis.

### Tirzepatide

In contrast to the GLP-1 receptor, the glucose-dependent insulinotropic polypeptide (GIP) receptor is present on adipocytes (Galindo et al., 2026[40]; Yip et al., 1998[159]). GIP is implicated in adipocyte carbohydrate and lipid metabolism through regulation of glucose uptake (Song et al., 2007[129] ), lipolysis (Getty-Kaushik et al., 2006[43]), and lipoprotein lipase activity (Miyawaki et al., 2002[89]). Tirzepatide, a GLP-1 receptor and GIP receptor co-agonist, has been approved for the treatment of T2DM and obesity and was not only found superior to GLP-1 receptor agonists to decrease bodyweight, both in people with and without T2DM, but also HbA1c levels in patients with diabetes (Campbell et al., 2023[20]). In a phase 2 trial of tirzepatide (5 mg, 10 mg, or 15 mg) subcutaneously once weekly for 52 weeks in 190 participants with MASH and stage F2 or F3 (moderate or severe) fibrosis, tirzepatide treatment led to statistically significant MASH resolution without worsening of fibrosis in 44 % of 5 mg, 56 % of 10 mg, and 62 % of 15 mg tirzepatide groups compared with 10 % in the placebo group (Loomba et al., 2024[80]). In this trial (SYNERGY-NASH trial), improvement of at least one fibrosis stage without worsening of MASH was found in 55 % of 5 mg, 51 % of 10 mg, and 51 % of the 15 mg tirzepatide groups compared with 30 % in the placebo group.

### Other GLP-1 based therapies

There are few RCTs of other GLP-1 receptor agonists in individuals with MASH proven by biopsy. In a phase 2 trial of liraglutide 1.8 mg subcutaneous once daily for 48 weeks, treatment with liraglutide led to MASH resolution in nine (39 %) of 23 patients compared with two (9 %) of 22 in the placebo group (relative risk 43, 95 % CI 1.0-17.7) (Armstrong et al., 2016[10]). No improvement of fibrosis, but a lower rate of worsening of fibrosis, was observed in the liraglutide group.

Survodutide is a dual GLP-1 and glucagon receptor agonist that is in development. Glucagon responds to hypoglycemia by mobilizing hepatic glucose through the stimulation of glycogenolysis and gluconeogenesis (Habegger et al., 2010[46]). Despite its glucose mobilizing effect, the broad catabolic and thermogenic nature of glucagon receptor agonism adds to its attractive portfolio as a weight loss therapeutic (Hope et al., 2021[51]). In particular, glucagon has been demonstrated to enhance hepatic lipid metabolism (Galsgaard et al., 2019[41]). In a phase 2 trial of survodutide (2.4 mg, 4.8 mg, or 6.0 mg) subcutaneously once weekly for 48 weeks in 293 participants with biopsy-confirmed MASH and fibrosis stage F1-F3, improvement in MASH with no worsening of fibrosis occurred in 47 % of the participants in the survodutide 2.4-mg group, 62 % of those in the 4.8-mg group, and 43 % of those in the 6.0-mg group, as compared with 14 % of those in the placebo group (P < 0.001 for the quadratic dose-response curve as best-fitting model) (Sanyal et al., 2024[119]). Improvement in fibrosis by at least one stage occurred in 34 % of 2.4 mg, 36 % of 4.8 mg, 34 % of 6.0 mg groups compared with 22 % in the placebo group.

Pemvidutide is another dual GLP-1 and glucagon receptor agonist that is in development. In the study of pemvidutide (1.2 mg, 1.8 mg, or 2.4 mg) subcutaneously once weekly for 12 weeks in 94 patients with a BMI ≥ 28.0 kg/m^2^ and liver fat content (LFC) ≥ 10 % by magnetic resonance imaging-proton density fat fraction (MRI-PDFF), relative reductions in LFC from baseline were 46.6 % (95 % CI -63.7 to -29.6), 68.5 % (95 % CI -84.4 to -52.5), and 57.1 % (95 % CI -76.1 to -38.1) for the pemvidutide 1.2 mg, 1.8 mg, and 2.4 mg groups, respectively, vs. 4.4 % (95 % CI -20.2 to 11.3) for the placebo group (p < 0.001 vs. placebo, all treatment groups) (Harrison et al., 2025[50]). The IMPACT trial is an ongoing 48-week international, randomized, double-blind, placebo-controlled, phase 2b trial of pemvidutide in patients with biopsy-confirmed MASH and fibrosis stage F2 or F3 (Noureddin et al., 2025[94]). At 24 weeks of treatment, MASH resolution without fibrosis worsening was observed in 18 (20 %) of 86 patients in the placebo group, 24 (58 %) of 41 patients in the 1.2 mg pemvidutide group (difference of 38 % [95 % CI 21-56]; p < 0.0001), and 45 (52 %) of 85 patients in the 1.8 mg pemvidutide group (difference of 32 % [95 % CI 19-46]; p < 0.0001). Fibrosis improvement without worsening of MASH was observed in 24 (28 %) of 86 patients in the placebo group, 13 (33 %) of 41 patients in the 1.2 mg pemvidutide group (difference of 5 % [95 % CI -13 to 22]; p = 0·59), and 30 (36 %) of 85 patients in the 1.8 mg pemvidutide group (difference of 8 % [95 % CI -6 to 22]; p = 0·27). At 24 weeks, pemvidutide treatment met the primary endpoint of MASH resolution without worsening of fibrosis but did not meet the other primary endpoint of fibrosis improvement without worsening of MASH. Because the IMPACT trial is ongoing, longer-duration results will be needed to confirm the efficacy of pemvidutide for MASH.

Retatrutide is a novel triple agonist of the GLP-1, GIP, and glucagon receptors. In a phase 2a trial of retatrutide (1, 4, 8 or 12 mg dose) subcutaneously once weekly for 48 weeks in 98 patients with MASLD and LFC ≥ 10 % by MRI-PDFF, the mean relative change from baseline in LFC at 24 weeks was -42.9 % (1 mg), -57.0 % (4 mg), -81.4 % (8 mg), -82.4 % (12 mg) and +0.3 % (placebo) (all P < 0.001 versus placebo) (Sanyal et al., 2024[121]). Liver biopsy results of retatrutide treatment are not available yet.

### Pioglitazone

Pioglitazone is a peroxisome proliferator-activated receptor (PPAR)-γ agonist that acts as insulin sensitizer and improves insulin and glucose parameters, modulates adipose tissue distribution while decreasing visceral fat and increasing subcutaneous fat, and reduces lipotoxicity in the liver. Several studies have shown histological improvement in steatosis and lobular inflammation after pioglitazone treatment (Aithal et al., 2008[4]; Promrat et al., 2004[105]). A meta-analysis showed an OR of 3.65 (95 % CI 2.32-5.74) for MASH resolution and 10.17 (95 % CI 2.8-36.5) for improvement in advanced fibrosis (stages F3-F4) compared with control groups (Musso et al., 2017[92]). Although pioglitazone has not been approved to treat MASLD, it is attractive because they offer potential benefit compared with a lack of histological benefit (or clinical trial data) from other oral glucose-lowering therapies in MASLD. Guidelines on the management of MASLD state that pioglitazone can be used to treat patients with MASH, with or without T2DM (Chalasani et al., 2018[22]; European Association for the Study of the et Liver al., 2024[38]).

### Sodium-glucose cotransporter 2 (SGLT2) inhibitors

Sodium-glucose co-transporter 2 (SGLT2) is predominant in the S1 and S2 segments of the renal proximal tubule and functions as a high-capacity glucose transporter, responsible for approximately 90 % of glucose reabsorption in the renal tubules (Dai et al., 2023[31]; Lee et al., 2025[72]). SGLT2 inhibitor leads to reduced glucose and sodium reabsorption by the renal tubules, resulting in an increased excretion of glucose, sodium, and water in the urine (Lee et al., 2025[72]). SGLT2 inhibitor promotes weight loss, which is an attractive property for the treatment of patients with MASLD. Although histological evaluation of the efficacy of SGLT2 inhibitors in MASLD/MASH patients is still lacking, in a systematic review of seven trials, treatment with SGLT2 inhibitors was associated with an improvement in LFC, aminotransferase levels, bodyweight, and HbA1c levels (Mantovani et al., 2022[84]). A large population study in South Korea has shown that SGLT2 inhibitors were associated with a higher likelihood of MASLD regression when compared with thiazolidinediones, dipeptidyl peptidase-4 inhibitors, and sulfonylureas (Jang et al., 2024[55]). Because SGLT2 inhibitors might be effective in treating MASLD/MASH, most probably via their glucose and bodyweight-lowering effects, further studies will be warranted to evaluate the efficacy of SGLT2 inhibitors.

### Drugs under investigation and future direction

Multiple therapeutic targets for MASLD/MASH are under active investigation, reflecting the disease's complex and heterogeneous pathophysiology. Agents in development include fibroblast growth factor 21 analogues, pan-peroxisome proliferator-activated receptor agonists, small interfering RNAs targeting HSD17B13 or PNPLA3, farnesoid X receptor agonists, stearoyl-CoA desaturase 1 antagonists, CC-chemokine receptor antagonists, and therapies modulating the gut microbiota (Zhang et al., 2025[165]).

In recent years, there has been growing interest among researchers in elucidating the role of epigenetic modifications in the treatment of MASLD. DNA methyltransferase (DNMT) inhibitors, histone deacetylases (HDAC) inhibitors, and miRNA inhibitors are under investigation with a focus on gene regulation (Zaiou and Joubert, 2025[164]). DNMT inhibitors (azacitidine and zebularine) ameliorated different metabolic biochemical parameters and increased insulin sensitivity, preventing the progression and development of MASLD (Pant et al., 2023[98]). Some new nucleoside DNMT inhibitors and nonnucleoside DNMT inhibitors, including hydralazine, procaine and MG98, have been identified and are currently being investigated (Hu et al., 2021[52]). HDAC inhibitors (trichostatin A, sodium butyrate, and MS-275) suppressed hepatocyte apoptosis and increase cell viability (Lei et al., 2010[73]). Inhibition of miRNA-34a activated fatty acid oxidation, inhibited hepatocyte steatosis, reduced hepatocyte reactive oxygen species levels and decreased gene expression of fibrogenesis (Zhu et al., 2023[168]). Additionally, inhibition of miRNA-21 and miRNA-103-3p offers a potential therapeutic strategy for treating MASLD (Ding et al., 2022[33]; Wang et al., 2019[153]).

Because MASLD results from the convergence of metabolic dysfunction, inflammation, lipotoxicity, and fibrogenesis, monotherapy is unlikely to achieve sustained disease modification in many patients. As in other chronic metabolic diseases, rational combination therapy targeting complementary pathways represents a promising future strategy, although robust clinical evidence for specific regimens is still evolving.

## Conclusion

The prevalence of MASLD is increasing worldwide, and appropriate management of MASLD patients will be crucial for global health care. Although considerable heterogeneity in the pathogenesis of MASLD exists, multiple factors, including increased hepatic de novo lipogenesis, adipose tissue dysfunction, genetic predisposition, epigenetic mechanism, and other related factors, have been associated with the development and progression of MASLD (Figure 4(Fig. 4)). Patients with MASLD can progress to cirrhosis and HCC and are at increased risk of developing T2DM and CVD. The fundamental principle of MASLD treatment is weight loss to decrease hepatic fat accumulation and delay the progression of inflammation and fibrosis. However, because it is not easy to achieve and maintain weight loss by only lifestyle modification, pharmacological treatment is necessary in patients with MASLD. Resmetirom and semaglutide are approved for the treatment of MASH with moderate to advanced fibrosis, and many drugs are under investigation. It is important to raise awareness of MASLD and promote appropriate management starting now.

## Notes

Soo Lim and Cheol-Young Park (Division of Endocrinology and Metabolism, Department of Internal Medicine, Kangbuk Samsung Hospital, Sungkyunkwan University School of Medicine, 29 Saemunan-ro, Jongno-gu, Seoul, South Korea (Postal code: 03181); M: 82-10-3397-6107; E-mail: cydoctor@skku.edu) contributed equally as corresponding author.

## Declaration

### Artificial Intelligence (AI) - assisted technology

No artificial intelligence was used in creating the content or figures of this manuscript.

### Conflict of interest

The authors declare that they have no conflict of interest.

## Figures and Tables

**Table 1:**
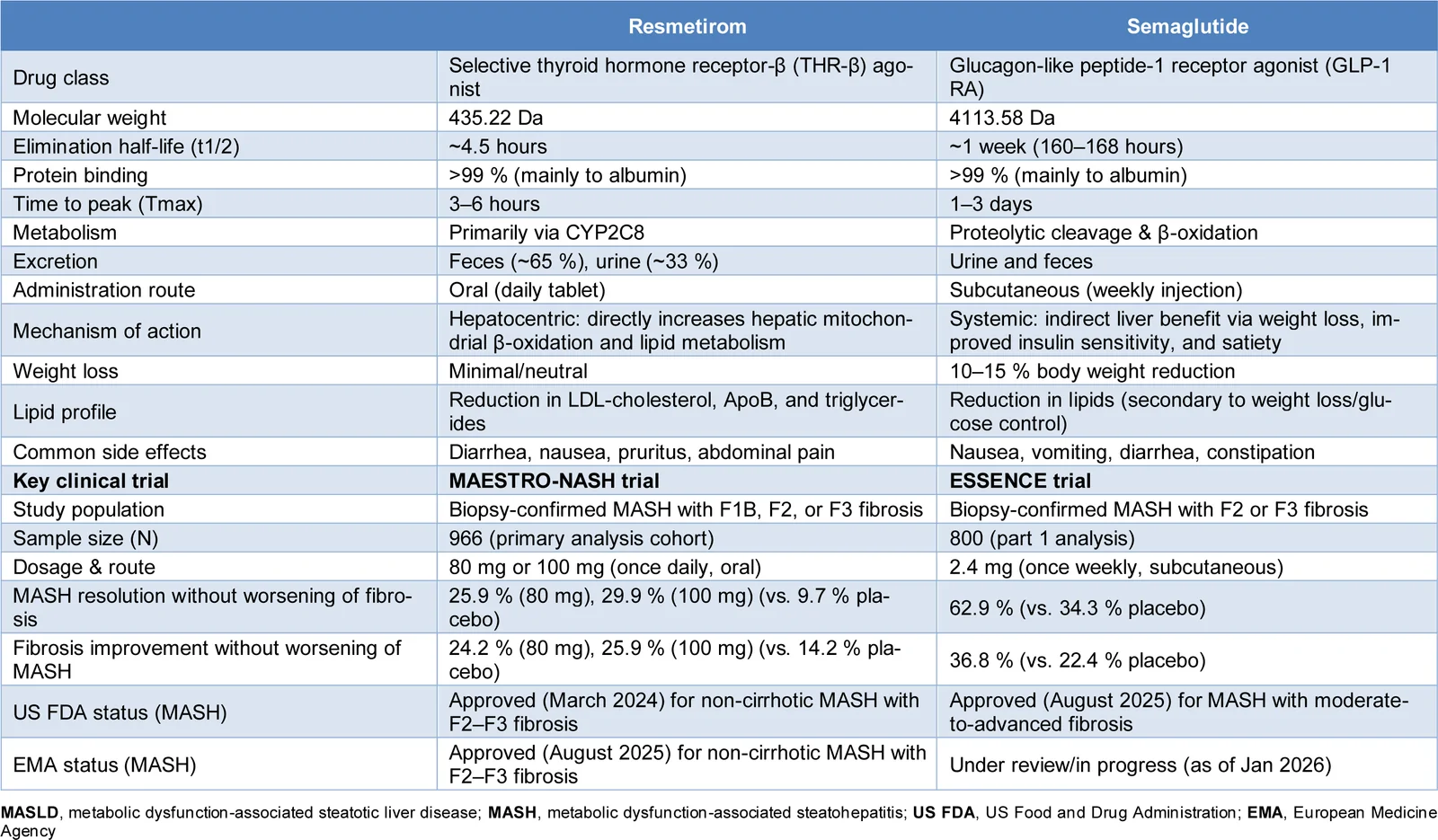
Comparison of resmetirom and semaglutide in MASLD/MASH

**Figure 1:**
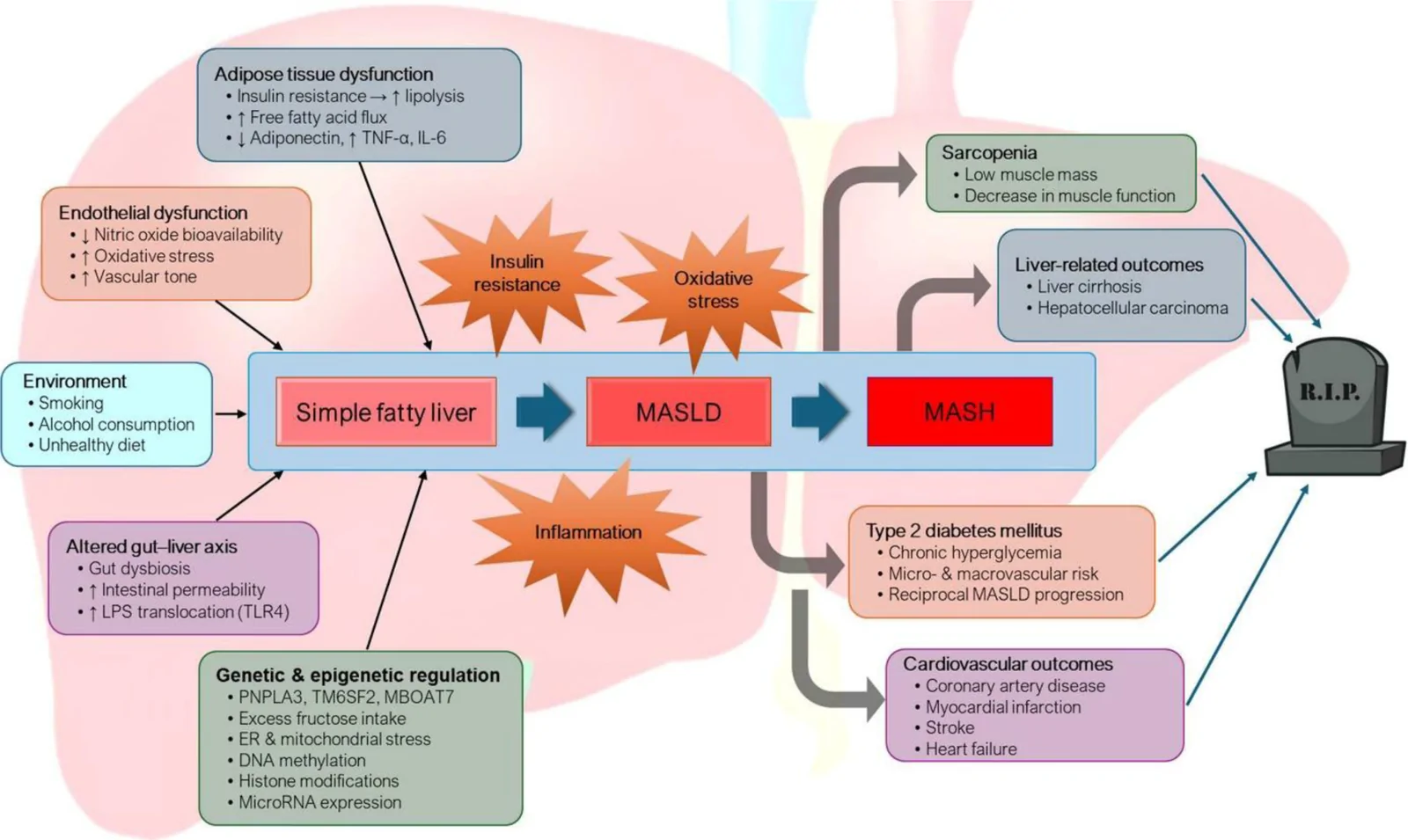
Graphical abstract

**Figure 2:**
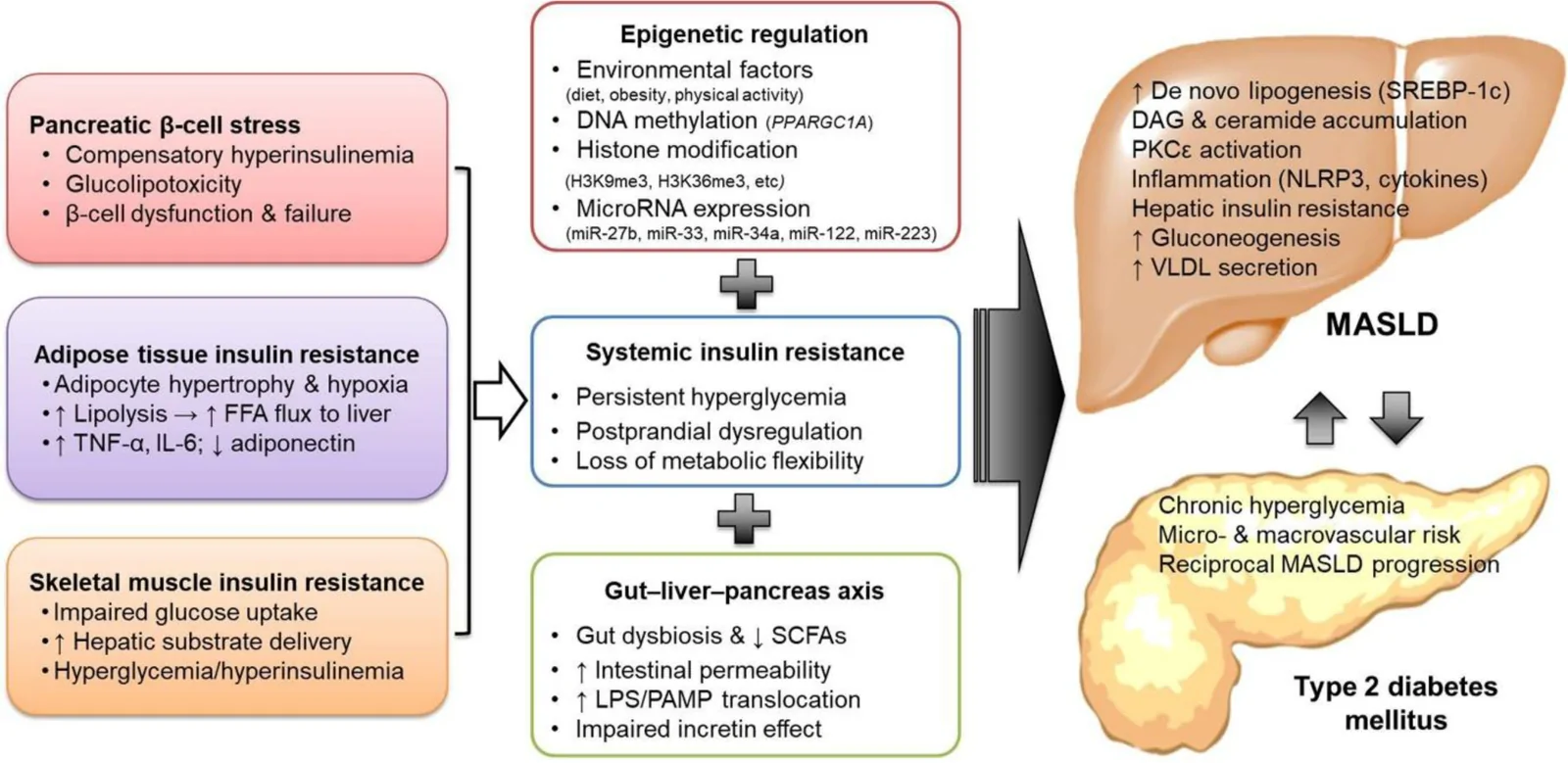
Pathophysiologic link between metabolic dysfunction-associated steatotic liver disease (MASLD) and type 2 diabetes mellitus (T2DM). Schematic illustration of the bidirectional interplay between MASLD and T2DM. Systemic insulin resistance arises from adipose tissue dysfunction (enhanced lipolysis and inflammation), skeletal muscle insulin resistance (impaired glucose uptake), and pancreatic β-cell stress (compensatory hyperinsulinemia and dysfunction). Epigenetic regulation (DNA methylation, histone modification, and microRNAs) influenced by environmental factors further modulates disease progression. Dysregulation of the gut-liver-pancreas axis contributes via gut dysbiosis, increased intestinal permeability, and endotoxin translocation. In the liver, increased de novo lipogenesis, lipid accumulation, inflammation, and hepatic insulin resistance promote gluconeogenesis and VLDL secretion. These processes drive chronic hyperglycemia, cardiometabolic risk, and reciprocal progression of MASLD and T2DM.

**Figure 3:**
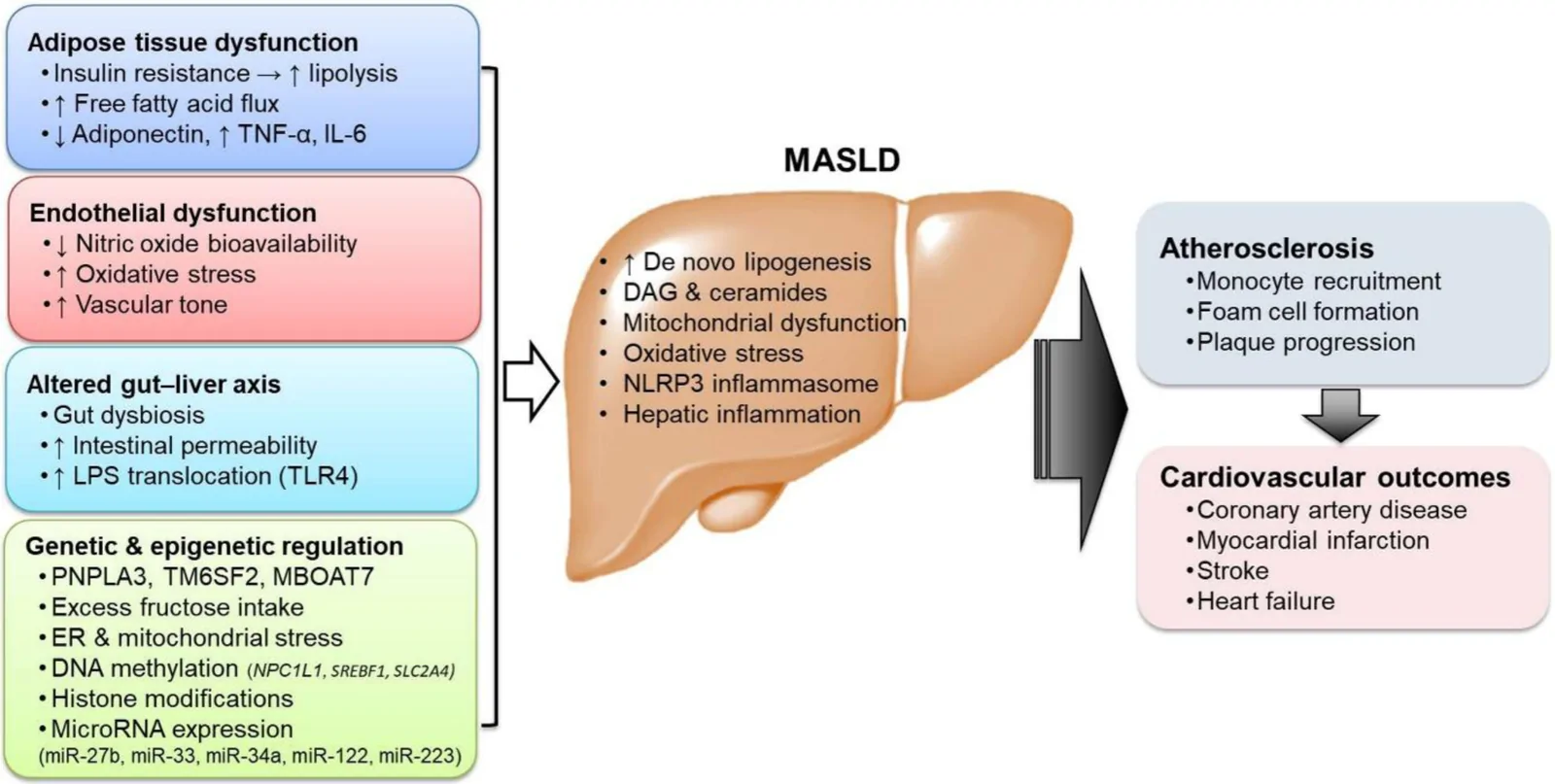
Pathophysiologic link between metabolic dysfunction-associated steatotic liver disease (MASLD) and cardiovascular disease (CVD). Schematic overview showing how adipose tissue dysfunction, endothelial dysfunction, gut-liver axis alterations, and genetic and epigenetic regulation contribute to hepatic lipotoxicity, including increased de novo lipogenesis, lipid accumulation, mitochondrial dysfunction, and inflammation. These hepatic changes promote systemic oxidative stress and vascular dysfunction, leading to a pro-atherogenic state characterized by monocyte recruitment, foam cell formation, and plaque progression, ultimately resulting in adverse cardiovascular outcomes such as coronary artery disease, myocardial infarction, stroke, and heart failure.

**Figure 4:**
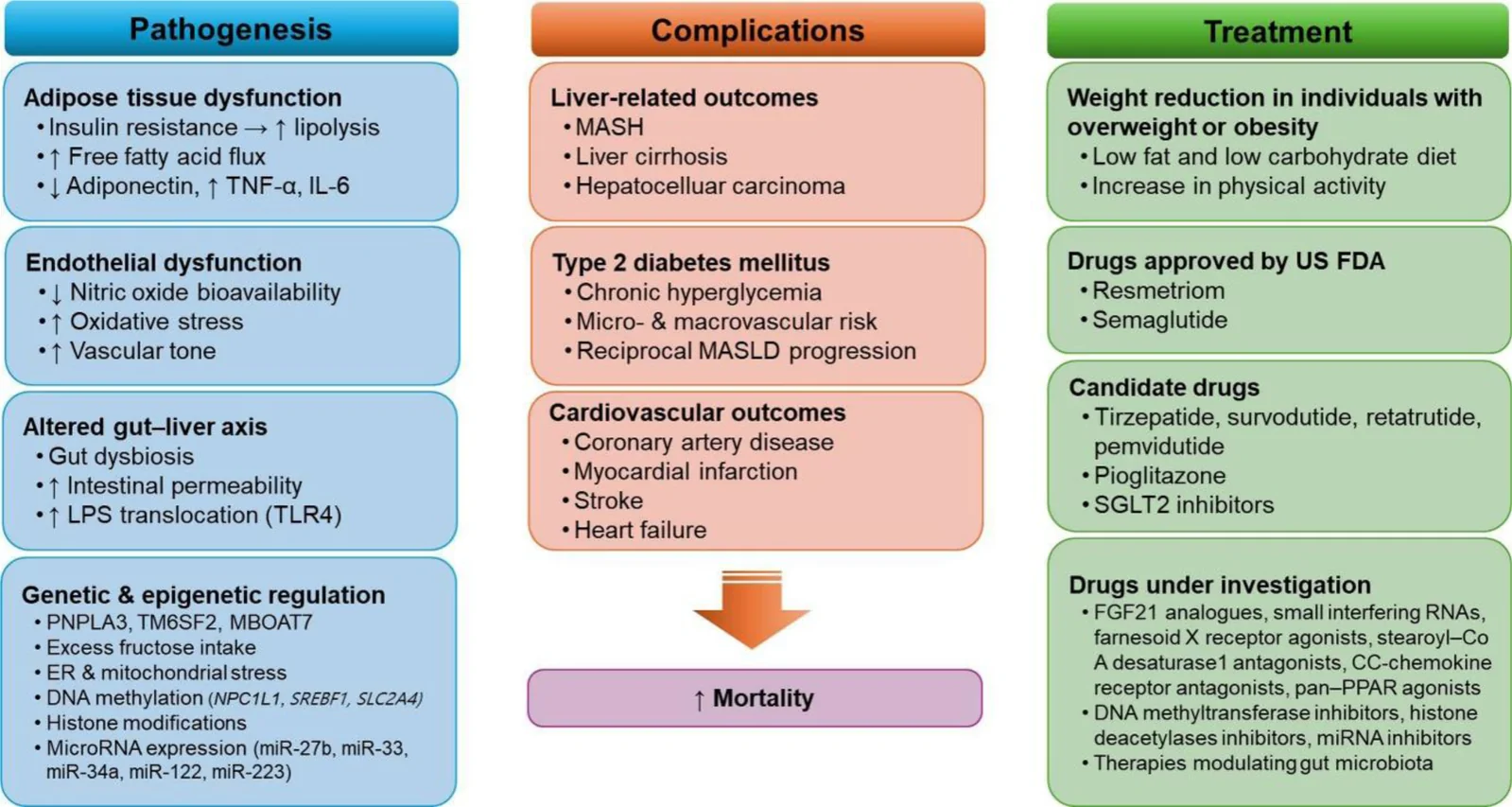
Pathogenesis, complications and treatment in metabolic dysfunction-associated steatotic liver disease (MASLD). Abbreviation: LPS, lipopolysaccharide; TLR4, toll-like receptor4; ER, endoplasmic reticulum; SGLT2, sodium-glucose cotransporter 2; FGF, fibroblast growth factor.

## References

[1] Abohashem S, Aldosoky W, Abbasi T, Dar T, Rollings R, Tung B (2026). Hepatic steatosis associates with leukopoietic activity and atherosclerotic inflammation: mechanistic insights into cardiovascular risk. Metabolism.

[2] Adams LA, Lymp JF, St Sauver J, Sanderson SO, Lindor KD, Feldstein A (2005). The natural history of nonalcoholic fatty liver disease: a population-based cohort study. Gastroenterology.

[3] Ahmed A, Cule M, Bell JD, Sattar N, Yaghootkar H (2024). Differing genetic variants associated with liver fat and their contrasting relationships with cardiovascular diseases and cancer. J Hepatol.

[4] Aithal GP, Thomas JA, Kaye PV, Lawson A, Ryder SD, Spendlove I (2008). Randomized, placebo-controlled trial of pioglitazone in nondiabetic subjects with nonalcoholic steatohepatitis. Gastroenterology.

[5] Ajmera V, Loomba R (2023). Advances in the genetics of nonalcoholic fatty liver disease. Curr Opin Gastroenterol.

[6] Alamuddin N, Wadden TA (2016). Behavioral Treatment of the Patient with Obesity. Endocrinol Metab Clin North Am.

[7] American Diabetes Association Professional Practice Committee for Diabetes (2026). 4. Comprehensive Medical Evaluation and Assessment of Comorbidities: Standards of Care in Diabetes-2026. Diabetes Care.

[8] Angulo P, Kleiner DE, Dam-Larsen S, Adams LA, Bjornsson ES, Charatcharoenwitthaya P (2015). Liver Fibrosis, but No Other Histologic Features, Is Associated With Long-term Outcomes of Patients With Nonalcoholic Fatty Liver Disease. Gastroenterology.

[9] Anstee QM, Targher G, Day CP (2013). Progression of NAFLD to diabetes mellitus, cardiovascular disease or cirrhosis. Nat Rev Gastroenterol Hepatol.

[10] Armstrong MJ, Gaunt P, Aithal GP, Barton D, Hull D, Parker R (2016). Liraglutide safety and efficacy in patients with non-alcoholic steatohepatitis (LEAN): a multicentre, double-blind, randomised, placebo-controlled phase 2 study. Lancet.

[11] Baffy G (2015). MicroRNAs in Nonalcoholic Fatty Liver Disease. J Clin Med.

[12] Ballestri S, Mantovani A, Baldelli E, Lugari S, Maurantonio M, Nascimbeni F (2021). Liver Fibrosis Biomarkers Accurately Exclude Advanced Fibrosis and Are Associated with Higher Cardiovascular Risk Scores in Patients with NAFLD or Viral Chronic Liver Disease. Diagnostics (Basel).

[13] Barone Gibbs B, Hivert MF, Jerome GJ, Kraus WE, Rosenkranz SK, Schorr EN (2021). Physical Activity as a Critical Component of First-Line Treatment for Elevated Blood Pressure or Cholesterol: Who, What, and How?: A Scientific Statement From the American Heart Association. Hypertension.

[14] Bartel DP (2004). MicroRNAs: genomics, biogenesis, mechanism, and function. Cell.

[15] Bedogni G, Bellentani S, Miglioli L, Masutti F, Passalacqua M, Castiglione A (2006). The Fatty Liver Index: a simple and accurate predictor of hepatic steatosis in the general population. BMC Gastroenterol.

[16] Biondi-Zoccai GG, Abbate A, Liuzzo G, Biasucci LM (2003). Atherothrombosis, inflammation, and diabetes. J Am Coll Cardiol.

[17] Bo T, Gao L, Yao Z, Shao S, Wang X, Proud CG (2024). Hepatic selective insulin resistance at the intersection of insulin signaling and metabolic dysfunction-associated steatotic liver disease. Cell Metab.

[18] Bril F, Cusi K (2016). Nonalcoholic Fatty Liver Disease: The New Complication of Type 2 Diabetes Mellitus. Endocrinol Metab Clin North Am.

[19] Brouwers M, Simons N, Stehouwer CDA, Isaacs A (2020). Non-alcoholic fatty liver disease and cardiovascular disease: assessing the evidence for causality. Diabetologia.

[20] Campbell JE, Muller TD, Finan B, DiMarchi RD, Tschop MH, D'Alessio DA (2023). GIPR/GLP-1R dual agonist therapies for diabetes and weight loss-chemistry, physiology, and clinical applications. Cell Metab.

[21] Castera L, Rinella ME, Tsochatzis EA (2025). Noninvasive Assessment of Liver Fibrosis. N Engl J Med.

[22] Chalasani N, Younossi Z, Lavine JE, Charlton M, Cusi K, Rinella M (2018). The diagnosis and management of nonalcoholic fatty liver disease: Practice guidance from the American Association for the Study of Liver Diseases. Hepatology.

[23] Chen VL, Morgan TR, Rotman Y, Patton HM, Cusi K, Kanwal F (2025). Resmetirom therapy for metabolic dysfunction-associated steatotic liver disease: October 2024 updates to AASLD Practice Guidance. Hepatology.

[24] Chen Y, Liang R, Li Y, Jiang L, Ma D, Luo Q (2024). Chromatin accessibility: biological functions, molecular mechanisms and therapeutic application. Signal Transduct Target Ther.

[25] Cheng WC, Chen HF, Cheng HC, Li CY (2024). Comparison of all-cause mortality associated with non-alcoholic fatty liver disease and metabolic dysfunction-associated fatty liver disease in Taiwan MJ cohort. Epidemiol Health.

[26] Cho Y, Lim SK, Joo SK, Jeong DH, Kim JH, Bae JM (2019). Nonalcoholic steatohepatitis is associated with a higher risk of advanced colorectal neoplasm. Liver Int.

[27] Chung GE, Yu SJ, Park J, Kim YJ, Yoon JH, Han K (2026). Associations between steatotic liver disease subtypes and incident diabetes in young Korean adults: A nationwide cohort study. Diabetes Obes Metab.

[28] Cotter TG, Rinella M (2020). Nonalcoholic Fatty Liver Disease 2020: The State of the Disease. Gastroenterology.

[29] Cusi K, Isaacs S, Barb D, Basu R, Caprio S, Garvey WT (2022). American Association of Clinical Endocrinology Clinical Practice Guideline for the Diagnosis and Management of Nonalcoholic Fatty Liver Disease in Primary Care and Endocrinology Clinical Settings: Co-Sponsored by the American Association for the Study of Liver Diseases (AASLD). Endocr Pract.

[30] Cuthbertson DJ, Weickert MO, Lythgoe D, Sprung VS, Dobson R, Shoajee-Moradie F (2014). External validation of the fatty liver index and lipid accumulation product indices, using 1H-magnetic resonance spectroscopy, to identify hepatic steatosis in healthy controls and obese, insulin-resistant individuals. Eur J Endocrinol.

[31] Dai ZC, Chen JX, Zou R, Liang XB, Tang JX, Yao CW (2023). Role and mechanisms of SGLT-2 inhibitors in the treatment of diabetic kidney disease. Front Immunol.

[32] Dimitrova E, Turberfield AH, Klose RJ (2015). Histone demethylases in chromatin biology and beyond. EMBO Rep.

[33] Ding J, Xia C, Cen P, Li S, Yu L, Zhu J (2022). MiR-103-3p promotes hepatic steatosis to aggravate nonalcoholic fatty liver disease by targeting of ACOX1. Mol Biol Rep.

[34] Donnelly KL, Smith CI, Schwarzenberg SJ, Jessurun J, Boldt MD, Parks EJ (2005). Sources of fatty acids stored in liver and secreted via lipoproteins in patients with nonalcoholic fatty liver disease. J Clin Invest.

[35] Drucker DJ (2018). Mechanisms of Action and Therapeutic Application of Glucagon-like Peptide-1. Cell Metab.

[36] En Li Cho E, Ang CZ, Quek J, Fu CE, Lim LKE, Heng ZEQ (2023). Global prevalence of non-alcoholic fatty liver disease in type 2 diabetes mellitus: an updated systematic review and meta-analysis. Gut.

[37] Erceg S, Ninic A, Kotur-Stevuljevic J, Ben Mariem O, Mitrovic M, Munjas J (2025). Transcriptomic, Redox Status and Adipocytokine Profiles in Metabolic Dysfunction-Associated Steatotic Liver Disease: Impact of Coexisting Type 2 Diabetes. Med Sci (Basel).

[38] European Association for the Study of the Liver, European Association for the Study of Diabetes, European Association for the Study of Obesity (2024). EASL-EASD-EASO Clinical Practice Guidelines on the management of metabolic dysfunction-associated steatotic liver disease (MASLD). J Hepatol.

[39] Friedman SL, Neuschwander-Tetri BA, Rinella M, Sanyal AJ (2018). Mechanisms of NAFLD development and therapeutic strategies. Nat Med.

[40] Galindo RJ, Cheng AYY, Longuet C, Ai M, Coskun T, Malik R (2026). Insights into the Mechanism of Action of Tirzepatide: A Narrative Review. Diabetes Ther.

[41] Galsgaard KD, Pedersen J, Knop FK, Holst JJ, Wewer Albrechtsen NJ (2019). Glucagon Receptor Signaling and Lipid Metabolism. Front Physiol.

[42] Gastaldelli A, Cusi K (2019). From NASH to diabetes and from diabetes to NASH: Mechanisms and treatment options. JHEP Rep.

[43] Getty-Kaushik L, Song DH, Boylan MO, Corkey BE, Wolfe MM (2006). Glucose-dependent insulinotropic polypeptide modulates adipocyte lipolysis and reesterification. Obesity (Silver Spring).

[44] Ghaben AL, Scherer PE (2019). Adipogenesis and metabolic health. Nat Rev Mol Cell Biol.

[45] Guo H, Pan L, Wu Q, Wang L, Huang Z, Wang J (2025). Type 2 Diabetes and the Multifaceted Gut-X Axes. Nutrients.

[46] Habegger KM, Heppner KM, Geary N, Bartness TJ, DiMarchi R, Tschop MH (2010). The metabolic actions of glucagon revisited. Nat Rev Endocrinol.

[47] Hagstrom H, Vessby J, Ekstedt M, Shang Y (2024). 99% of patients with NAFLD meet MASLD criteria and natural history is therefore identical. J Hepatol.

[48] Han E, Han KD, Lee YH, Kim KS, Hong S, Park JH (2025). Association of temporal MASLD with type 2 diabetes, cardiovascular disease and mortality. Cardiovasc Diabetol.

[49] Harrison SA, Bedossa P, Guy CD, Schattenberg JM, Loomba R, Taub R (2024). A Phase 3, Randomized, Controlled Trial of Resmetirom in NASH with Liver Fibrosis. N Engl J Med.

[50] Harrison SA, Browne SK, Suschak JJ, Tomah S, Gutierrez JA, Yang J (2025). Effect of pemvidutide, a GLP-1/glucagon dual receptor agonist, on MASLD: A randomized, double-blind, placebo-controlled study. J Hepatol.

[51] Hope DCD, Vincent ML, Tan TMM (2021). Striking the Balance: GLP-1/Glucagon Co-Agonism as a Treatment Strategy for Obesity. Front Endocrinol (Lausanne).

[52] Hu C, Liu X, Zeng Y, Liu J, Wu F (2021). DNA methyltransferase inhibitors combination therapy for the treatment of solid tumor: mechanism and clinical application. Clin Epigenetics.

[53] Huang DQ, Noureddin N, Ajmera V, Amangurbanova M, Bettencourt R, Truong E (2023). Type 2 diabetes, hepatic decompensation, and hepatocellular carcinoma in patients with non-alcoholic fatty liver disease: an individual participant-level data meta-analysis. Lancet Gastroenterol Hepatol.

[54] Huang X, Xu M, Chen Y, Peng K, Huang Y, Wang P (2015). Validation of the Fatty Liver Index for Nonalcoholic Fatty Liver Disease in Middle-Aged and Elderly Chinese. Medicine (Baltimore).

[55] Jang H, Kim Y, Lee DH, Joo SK, Koo BK, Lim S (2024). Outcomes of Various Classes of Oral Antidiabetic Drugs on Nonalcoholic Fatty Liver Disease. JAMA Intern Med.

[56] Jing M, Jiang Y (2025). Microbiome-mediated crosstalk between T2DM and MASLD: a translational review focused on function. Front Endocrinol (Lausanne).

[57] Johnson SM, Bao H, McMahon CE, Chen Y, Burr SD, Anderson AM (2024). PNPLA3 is a triglyceride lipase that mobilizes polyunsaturated fatty acids to facilitate hepatic secretion of large-sized very low-density lipoprotein. Nat Commun.

[58] Kahn CR, Wang G, Lee KY (2019). Altered adipose tissue and adipocyte function in the pathogenesis of metabolic syndrome. J Clin Invest.

[59] Kanwal F, Shubrook JH, Adams LA, Pfotenhauer K, Wai-Sun Wong V, Wright E (2021). Clinical Care Pathway for the Risk Stratification and Management of Patients With Nonalcoholic Fatty Liver Disease. Gastroenterology.

[60] Karpe F, Pinnick KE (2015). Biology of upper-body and lower-body adipose tissue--link to whole-body phenotypes. Nat Rev Endocrinol.

[61] Kim D, Konyn P, Sandhu KK, Dennis BB, Cheung AC, Ahmed A (2021). Metabolic dysfunction-associated fatty liver disease is associated with increased all-cause mortality in the United States. J Hepatol.

[62] Kim KS, Hong S, Ahn HY, Park CY (2023). Metabolic Dysfunction-Associated Fatty Liver Disease and Mortality: A Population-Based Cohort Study. Diabetes Metab J.

[63] Kim KS, Hong S, Han K, Park CY (2024). Association of non-alcoholic fatty liver disease with cardiovascular disease and all cause death in patients with type 2 diabetes mellitus: nationwide population based study. BMJ.

[64] Kim KS, Lee BW (2020). Beneficial effect of anti-diabetic drugs for nonalcoholic fatty liver disease. Clin Mol Hepatol.

[65] Kim KS, Lee BW, Kim YJ, Lee DH, Cha BS, Park CY (2019). Nonalcoholic Fatty Liver Disease and Diabetes: Part II: Treatment. Diabetes Metab J.

[66] Kokkorakis M, Boutari C, Hill MA, Kotsis V, Loomba R, Sanyal AJ (2024). Resmetirom, the first approved drug for the management of metabolic dysfunction-associated steatohepatitis: Trials, opportunities, and challenges. Metabolism.

[67] Korenblat KM, Fabbrini E, Mohammed BS, Klein S (2008). Liver, muscle, and adipose tissue insulin action is directly related to intrahepatic triglyceride content in obese subjects. Gastroenterology.

[68] Landrier JF, Derghal A, Mounien L (2019). MicroRNAs in Obesity and Related Metabolic Disorders. Cells.

[69] Lauridsen BK, Stender S, Kristensen TS, Kofoed KF, Kober L, Nordestgaard BG (2018). Liver fat content, non-alcoholic fatty liver disease, and ischaemic heart disease: Mendelian randomization and meta-analysis of 279 013 individuals. Eur Heart J.

[70] Lazo M, Hernaez R, Bonekamp S, Kamel IR, Brancati FL, Guallar E (2011). Non-alcoholic fatty liver disease and mortality among US adults: prospective cohort study. BMJ.

[71] Lee YH, Cho Y, Lee BW, Park CY, Lee DH, Cha BS (2019). Nonalcoholic Fatty Liver Disease in Diabetes. Part I: Epidemiology and Diagnosis. Diabetes Metab J.

[72] Lee YH, Lim S, Davies MJ (2025). Cardiometabolic and renal benefits of sodium-glucose cotransporter 2 inhibitors. Nat Rev Endocrinol.

[73] Lei WW, Zhang KH, Pan XC, Wang DM, Hu Y, Yang YN (2010). Histone deacetylase 1 and 2 differentially regulate apoptosis by opposing effects on extracellular signal-regulated kinase 1/2. Cell Death Dis.

[74] Liang J, Chu Y, Chen X, Leng Y (2025). The gut-adipose/pancreas axis: a novel perspective on glycolipid metabolism dysregulation in MAFLD and T2DM pathogenesis. Front Endocrinol (Lausanne).

[75] Lichtenstein AH, Appel LJ, Vadiveloo M, Hu FB, Kris-Etherton PM, Rebholz CM (2021). 2021 Dietary Guidance to Improve Cardiovascular Health: A Scientific Statement From the American Heart Association. Circulation.

[76] Lim S, Taskinen MR, Boren J (2019). Crosstalk between nonalcoholic fatty liver disease and cardiometabolic syndrome. Obes Rev.

[77] Liu CH, Zeng QM, Kim W, Kim SU, Younossi ZM, Targher G (2026). Sarcopenia and MASLD: novel insights and the future. Nat Rev Endocrinol.

[78] Long W, Hui Ju Z, Fan Z, Jing W, Qiong L (2014). The effect of recombinant adeno-associated virus-adiponectin (rAAV2/1-Acrp30) on glycolipid dysmetabolism and liver morphology in diabetic rats. Gen Comp Endocrinol.

[79] Loomba R, Friedman SL, Shulman GI (2021). Mechanisms and disease consequences of nonalcoholic fatty liver disease. Cell.

[80] Loomba R, Hartman ML, Lawitz EJ, Vuppalanchi R, Boursier J, Bugianesi E (2024). Tirzepatide for Metabolic Dysfunction-Associated Steatohepatitis with Liver Fibrosis. N Engl J Med.

[81] Luukkonen PK, Porthan K, Ahlholm N, Rosqvist F, Dufour S, Zhang XM (2023). The PNPLA3 I148M variant increases ketogenesis and decreases hepatic de novo lipogenesis and mitochondrial function in humans. Cell Metab.

[82] Lv X, Lv Y, Dai X (2023). Lactate, histone lactylation and cancer hallmarks. Expert Rev Mol Med.

[83] Mantovani A, Byrne CD, Bonora E, Targher G (2018). Nonalcoholic Fatty Liver Disease and Risk of Incident Type 2 Diabetes: A Meta-analysis. Diabetes Care.

[84] Mantovani A, Byrne CD, Targher G (2022). Efficacy of peroxisome proliferator-activated receptor agonists, glucagon-like peptide-1 receptor agonists, or sodium-glucose cotransporter-2 inhibitors for treatment of non-alcoholic fatty liver disease: a systematic review. Lancet Gastroenterol Hepatol.

[85] Mantovani A, Csermely A, Petracca G, Beatrice G, Corey KE, Simon TG (2021). Non-alcoholic fatty liver disease and risk of fatal and non-fatal cardiovascular events: an updated systematic review and meta-analysis. Lancet Gastroenterol Hepatol.

[86] Mantovani A, Petracca G, Beatrice G, Tilg H, Byrne CD, Targher G (2020). Non-alcoholic fatty liver disease and risk of incident diabetes mellitus: an updated meta-analysis of 501 022 adult individuals. Gut.

[87] Mantovani A, Petracca G, Csermely A, Beatrice G, Bonapace S, Rossi A (2022). Non-alcoholic fatty liver disease and risk of new-onset heart failure: an updated meta-analysis of about 11 million individuals. Gut.

[88] Miller RA, Chu Q, Le Lay J, Scherer PE, Ahima RS, Kaestner KH (2011). Adiponectin suppresses gluconeogenic gene expression in mouse hepatocytes independent of LKB1-AMPK signaling. J Clin Invest.

[89] Miyawaki K, Yamada Y, Ban N, Ihara Y, Tsukiyama K, Zhou H (2002). Inhibition of gastric inhibitory polypeptide signaling prevents obesity. Nat Med.

[90] Mohiuddin MS, Neha NT, Mahir JUK, Shakib FAF, Alam MA, Wahiduzzaman M (2025). Metabolic dysfunction associated fatty liver disease and type 2 diabetes: pathophysiological links, epidemiological trends, and clinical implications. Front Endocrinol (Lausanne).

[91] Moon JH, Kim W, Koo BK, Cho NH, Innovative Target Exploration of Nc (2022). Metabolic Dysfunction-Associated Fatty Liver Disease Predicts Long-term Mortality and Cardiovascular Disease. Gut Liver.

[92] Musso G, Cassader M, Paschetta E, Gambino R (2017). Thiazolidinediones and Advanced Liver Fibrosis in Nonalcoholic Steatohepatitis: A Meta-analysis. JAMA Intern Med.

[93] Mwinyi J, Bostrom AE, Pisanu C, Murphy SK, Erhart W, Schafmayer C (2017). NAFLD is associated with methylation shifts with relevance for the expression of genes involved in lipoprotein particle composition. Biochim Biophys Acta Mol Cell Biol Lipids.

[94] Noureddin M, Harrison SA, Loomba R, Alkhouri N, Chalasani N, Sheikh MY (2025). Safety and efficacy of weekly pemvidutide versus placebo for metabolic dysfunction-associated steatohepatitis (IMPACT): 24-week results from a multicentre, randomised, double-blind, phase 2b study. Lancet.

[95] Oki M, Aihara H, Ito T (2007). Role of histone phosphorylation in chromatin dynamics and its implications in diseases. Subcell Biochem.

[96] Ong JP, Pitts A, Younossi ZM (2008). Increased overall mortality and liver-related mortality in non-alcoholic fatty liver disease. J Hepatol.

[97] Pandey H, Goel P, Srinivasan VM, Tang DWT, Wong SH, Lal D (2025). Gut microbiota in non-alcoholic fatty liver disease: Pathophysiology, diagnosis, and therapeutics. World J Hepatol.

[98] Pant R, Kabeer SW, Sharma S, Kumar V, Patra D, Pal D (2023). Pharmacological inhibition of DNMT1 restores macrophage autophagy and M2 polarization in Western diet-induced nonalcoholic fatty liver disease. J Biol Chem.

[99] Pearce SR, Mimmack ML, Gallagher MP, Gileadi U, Hyde SC, Higgins CF (1992). Membrane topology of the integral membrane components, OppB and OppC, of the oligopeptide permease of Salmonella typhimurium. Mol Microbiol.

[100] Pirola CJ, Sookoian S (2022). Epigenetics factors in nonalcoholic fatty liver disease. Expert Rev Gastroenterol Hepatol.

[101] Pisto P, Santaniemi M, Bloigu R, Ukkola O, Kesaniemi YA (2014). Fatty liver predicts the risk for cardiovascular events in middle-aged population: a population-based cohort study. BMJ Open.

[102] Polyzos SA, Kountouras J, Mantzoros CS (2016). Adipokines in nonalcoholic fatty liver disease. Metabolism.

[103] Powell EE, Wong VW, Rinella M (2021). Non-alcoholic fatty liver disease. Lancet.

[104] Promrat K, Kleiner DE, Niemeier HM, Jackvony E, Kearns M, Wands JR (2010). Randomized controlled trial testing the effects of weight loss on nonalcoholic steatohepatitis. Hepatology.

[105] Promrat K, Lutchman G, Uwaifo GI, Freedman RJ, Soza A, Heller T (2004). A pilot study of pioglitazone treatment for nonalcoholic steatohepatitis. Hepatology.

[106] Rao S, Pandey A, Garg S, Park B, Mayo H, Despres JP (2019). Effect of Exercise and Pharmacological Interventions on Visceral Adiposity: A Systematic Review and Meta-analysis of Long-term Randomized Controlled Trials. Mayo Clin Proc.

[107] Ratti C, Malaguti M, Emanuele D, Bellasi A, Sanna G (2025). Understanding MASLD - from molecular pathogenesis to cardiovascular risk: A concise review for the clinical cardiologist. Atherosclerosis.

[108] Ratziu V, Boursier J, AFEF Group for the Study of Liver Fibrosis (2024). Confirmatory biomarker diagnostic studies are not needed when transitioning from NAFLD to MASLD. J Hepatol.

[109] Riazi K, Azhari H, Charette JH, Underwood FE, King JA, Afshar EE (2022). The prevalence and incidence of NAFLD worldwide: a systematic review and meta-analysis. Lancet Gastroenterol Hepatol.

[110] Rinella ME (2015). Nonalcoholic fatty liver disease: a systematic review. JAMA.

[111] Rinella ME, Lazarus JV, Ratziu V, Francque SM, Sanyal AJ, Kanwal F (2023). A multisociety Delphi consensus statement on new fatty liver disease nomenclature. Hepatology.

[112] Rinella ME, Neuschwander-Tetri BA, Siddiqui MS, Abdelmalek MF, Caldwell S, Barb D (2023). AASLD Practice Guidance on the clinical assessment and management of nonalcoholic fatty liver disease. Hepatology.

[113] Roden M, Shulman GI (2019). The integrative biology of type 2 diabetes. Nature.

[114] Rodriguez-Sanabria JS, Escutia-Gutierrez R, Rosas-Campos R, Armendariz-Borunda JS, Sandoval-Rodriguez A (2021). An Update in Epigenetics in Metabolic-Associated Fatty Liver Disease. Front Med (Lausanne).

[115] Romeo S, Sanyal A, Valenti L (2020). Leveraging Human Genetics to Identify Potential New Treatments for Fatty Liver Disease. Cell Metab.

[116] Rosenberg WM, Voelker M, Thiel R, Becka M, Burt A, Schuppan D (2004). Serum markers detect the presence of liver fibrosis: a cohort study. Gastroenterology.

[117] Santos JL, Krause BJ, Cataldo LR, Vega J, Salas-Pérez F, Mennickent P (2020). PPARGC1A Gene Promoter Methylation as a Biomarker of Insulin Secretion and Sensitivity in Response to Glucose Challenges. Nutrients.

[118] Santos RD, Valenti L, Romeo S (2019). Does nonalcoholic fatty liver disease cause cardiovascular disease? Current knowledge and gaps. Atherosclerosis.

[119] Sanyal AJ, Bedossa P, Fraessdorf M, Neff GW, Lawitz E, Bugianesi E (2024). A Phase 2 Randomized Trial of Survodutide in MASH and Fibrosis. N Engl J Med.

[120] Sanyal AJ, Brunt EM, Kleiner DE, Kowdley KV, Chalasani N, Lavine JE (2011). Endpoints and clinical trial design for nonalcoholic steatohepatitis. Hepatology.

[121] Sanyal AJ, Kaplan LM, Frias JP, Brouwers B, Wu Q, Thomas MK (2024). Triple hormone receptor agonist retatrutide for metabolic dysfunction-associated steatotic liver disease: a randomized phase 2a trial. Nat Med.

[122] Sanyal AJ, Newsome PN, Kliers I, Ostergaard LH, Long MT, Kjaer MS (2025). Phase 3 Trial of Semaglutide in Metabolic Dysfunction-Associated Steatohepatitis. N Engl J Med.

[123] Sanyal AJ, Van Natta ML, Clark J, Neuschwander-Tetri BA, Diehl A, Dasarathy S (2021). Prospective Study of Outcomes in Adults with Nonalcoholic Fatty Liver Disease. N Engl J Med.

[124] Sheka AC, Adeyi O, Thompson J, Hameed B, Crawford PA, Ikramuddin S (2020). Nonalcoholic Steatohepatitis: A Review. JAMA.

[125] Shulman GI (2014). Ectopic fat in insulin resistance, dyslipidemia, and cardiometabolic disease. N Engl J Med.

[126] Sinha RA, Bruinstroop E, Singh BK, Yen PM (2019). Nonalcoholic Fatty Liver Disease and Hypercholesterolemia: Roles of Thyroid Hormones, Metabolites, and Agonists. Thyroid.

[127] Smith GI, Shankaran M, Yoshino M, Schweitzer GG, Chondronikola M, Beals JW (2020). Insulin resistance drives hepatic de novo lipogenesis in nonalcoholic fatty liver disease. J Clin Invest.

[128] Son JW, le Roux CW, Bluher M, Nauck MA, Lim S (2026). Novel GLP-1-based Medications for Type 2 Diabetes and Obesity. Endocr Rev.

[129] Song DH, Getty-Kaushik L, Tseng E, Simon J, Corkey BE, Wolfe MM (2007). Glucose-dependent insulinotropic polypeptide enhances adipocyte development and glucose uptake in part through Akt activation. Gastroenterology.

[130] Song R, Li Z, Zhang Y, Tan J, Chen Z (2024). Comparison of NAFLD, MAFLD and MASLD characteristics and mortality outcomes in United States adults. Liver Int.

[131] Song SJ, Lai JC, Wong GL, Wong VW, Yip TC (2024). Can we use old NAFLD data under the new MASLD definition?. J Hepatol.

[132] Sookoian S, Rosselli MS, Gemma C, Burgueno AL, Fernandez Gianotti T, Castano GO (2010). Epigenetic regulation of insulin resistance in nonalcoholic fatty liver disease: impact of liver methylation of the peroxisome proliferator-activated receptor gamma coactivator 1alpha promoter. Hepatology.

[133] Stefan N (2020). Causes, consequences, and treatment of metabolically unhealthy fat distribution. Lancet Diabetes Endocrinol.

[134] Stefan N, Schick F, Haring HU (2017). Causes, Characteristics, and Consequences of Metabolically Unhealthy Normal Weight in Humans. Cell Metab.

[135] Stefan N, Yki-Järvinen H, Neuschwander-Tetri BA (2025). Metabolic dysfunction-associated steatotic liver disease: heterogeneous pathomechanisms and effectiveness of metabolism-based treatment. Lancet Diabetes Endocrinol.

[136] Sterling RK, Lissen E, Clumeck N, Sola R, Correa MC, Montaner J (2006). Development of a simple noninvasive index to predict significant fibrosis in patients with HIV/HCV coinfection. Hepatology.

[137] Szabo G, Csak T (2016). Role of MicroRNAs in NAFLD/NASH. Dig Dis Sci.

[138] Tan M, Luo H, Lee S, Jin F, Yang JS, Montellier E (2011). Identification of 67 histone marks and histone lysine crotonylation as a new type of histone modification. Cell.

[139] Targher G, Byrne CD, Lonardo A, Zoppini G, Barbui C (2016). Non-alcoholic fatty liver disease and risk of incident cardiovascular disease: A meta-analysis. J Hepatol.

[140] Targher G, Byrne CD, Tilg H (2024). MASLD: a systemic metabolic disorder with cardiovascular and malignant complications. Gut.

[141] Targher G, Corey KE, Byrne CD, Roden M (2021). The complex link between NAFLD and type 2 diabetes mellitus - mechanisms and treatments. Nat Rev Gastroenterol Hepatol.

[142] Targher G, Valenti L, Byrne CD (2025). Metabolic Dysfunction-Associated Steatotic Liver Disease. N Engl J Med.

[143] Taylor RS, Taylor RJ, Bayliss S, Hagström H, Nasr P, Schattenberg JM (2020). Association Between Fibrosis Stage and Outcomes of Patients With Nonalcoholic Fatty Liver Disease: A Systematic Review and Meta-Analysis. Gastroenterology.

[144] Tchkonia T, Thomou T, Zhu Y, Karagiannides I, Pothoulakis C, Jensen MD (2013). Mechanisms and metabolic implications of regional differences among fat depots. Cell Metab.

[145] Thoma C, Day CP, Trenell MI (2012). Lifestyle interventions for the treatment of non-alcoholic fatty liver disease in adults: a systematic review. J Hepatol.

[146] Tilg H, Moschen AR (2010). Evolution of inflammation in nonalcoholic fatty liver disease: the multiple parallel hits hypothesis. Hepatology.

[147] Tilg H, Petta S, Stefan N, Targher G (2026). Metabolic Dysfunction-Associated Steatotic Liver Disease in Adults: A Review. JAMA.

[148] Vilar-Gomez E, Martinez-Perez Y, Calzadilla-Bertot L, Torres-Gonzalez A, Gra-Oramas B, Gonzalez-Fabian L (2015). Weight Loss Through Lifestyle Modification Significantly Reduces Features of Nonalcoholic Steatohepatitis. Gastroenterology.

[149] Wadden TA, Tronieri JS, Butryn ML (2020). Lifestyle modification approaches for the treatment of obesity in adults. Am Psychol.

[150] Wai CT, Greenson JK, Fontana RJ, Kalbfleisch JD, Marrero JA, Conjeevaram HS (2003). A simple noninvasive index can predict both significant fibrosis and cirrhosis in patients with chronic hepatitis C. Hepatology.

[151] Wang H, Wang L, Erdjument-Bromage H, Vidal M, Tempst P, Jones RS (2004). Role of histone H2A ubiquitination in Polycomb silencing. Nature.

[152] Wang L, Wang H, Wu J, Ji C, Wang Y, Gu M (2025). Gut microbiota and metabolomics in metabolic dysfunction-associated fatty liver disease: interaction, mechanism, and therapeutic value. Front Cell Infect Microbiol.

[153] Wang XM, Wang XY, Huang YM, Chen X, Lu MH, Shi L (2019). Role and mechanisms of action of microRNA‑21 as regards the regulation of the WNT/beta‑catenin signaling pathway in the pathogenesis of non‑alcoholic fatty liver disease. Int J Mol Med.

[154] Wang Y, Chen S, Tian C, Wang Q, Yang Z, Che W (2024). Association of systemic immune biomarkers with metabolic dysfunction-associated steatotic liver disease: a cross-sectional study of NHANES 2007-2018. Front Nutr.

[155] Wewege MA, Thom JM, Rye KA, Parmenter BJ (2018). Aerobic, resistance or combined training: A systematic review and meta-analysis of exercise to reduce cardiovascular risk in adults with metabolic syndrome. Atherosclerosis.

[156] Wu YL, Lin ZJ, Li CC, Lin X, Shan SK, Guo B (2023). Epigenetic regulation in metabolic diseases: mechanisms and advances in clinical study. Signal Transduct Target Ther.

[157] Xia JK, Qin XQ, Zhang L, Liu SJ, Shi XL, Ren HZ (2022). Roles and regulation of histone acetylation in hepatocellular carcinoma. Front Genet.

[158] Xue Y, Peng Y, Zhang L, Ba Y, Jin G, Liu G (2024). Effect of different exercise modalities on nonalcoholic fatty liver disease: a systematic review and network meta-analysis. Sci Rep.

[159] Yip RG, Boylan MO, Kieffer TJ, Wolfe MM (1998). Functional GIP receptors are present on adipocytes. Endocrinology.

[160] Yki-Järvinen H, Luukkonen PK, Hodson L, Moore JB (2021). Dietary carbohydrates and fats in nonalcoholic fatty liver disease. Nat Rev Gastroenterol Hepatol.

[161] Younossi ZM, Golabi P, Paik JM, Henry A, Van Dongen C, Henry L (2023). The global epidemiology of nonalcoholic fatty liver disease (NAFLD) and nonalcoholic steatohepatitis (NASH): a systematic review. Hepatology.

[162] Younossi ZM, Henry L, Isaacs S, Cusi K (2023). Identification of High-Risk Patients With Nonalcoholic Fatty Liver Disease in Endocrinology Clinics. Endocr Pract.

[163] Younossi ZM, Zelber-Sagi S, Henry L, Gerber LH (2023). Lifestyle interventions in nonalcoholic fatty liver disease. Nat Rev Gastroenterol Hepatol.

[164] Zaiou M, Joubert O (2025). Racial and Ethnic Disparities in NAFLD: Harnessing Epigenetic and Gut Microbiota Pathways for Targeted Therapeutic Approaches. Biomolecules.

[165] Zhang X, Lau HC, Yu J (2025). Pharmacological treatment for metabolic dysfunction-associated steatotic liver disease and related disorders: Current and emerging therapeutic options. Pharmacol Rev.

[166] Zhou XD, Chen QF, Fan QY, Kim SU, Yip TC, Petta S (2026). Cardiovascular-Kidney-Metabolic syndrome and the risk of liver fibrosis progression and liver-related events in MASLD. Hepatology.

[167] Zhou YY, Zhou XD, Wu SJ, Fan DH, Van Poucke S, Chen YP (2018). Nonalcoholic fatty liver disease contributes to subclinical atherosclerosis: A systematic review and meta-analysis. Hepatol Commun.

[168] Zhu Y, Tan JK, Wong SK, Goon JA (2023). Therapeutic Effects of microRNAs on Nonalcoholic Fatty Liver Disease (NAFLD) and Nonalcoholic Steatohepatitis (NASH): A Systematic Review and Meta-Analysis. Int J Mol Sci.

